# Surgical starting time of the day and survival in gastric cancer

**DOI:** 10.1038/s41598-023-33692-0

**Published:** 2023-04-28

**Authors:** Yunhe Gao, Hongqing Xi, Fredrik Mattsson, Wenquan Liang, Shao-Hua Xie, Lin Chen, Jesper Lagergren

**Affiliations:** 1 Department of General Surgery, Chinese People’s Liberation Army General Hospital, Fuxing Road 28, Beijing, People’s Republic of China; 2 Upper Gastrointestinal Surgery, Department of Molecular Medicine and Surgery, Karolinska Institutet, Karolinska University Hospital, Retzius väg 13 a, 171 77 Stockholm, Sweden; 3 School of Cancer and Pharmaceutical Sciences, King’s College London, London, UK; 4 Institute of Population Medicine and School of Public Health, Fujian Medical University, Fuzhou, People’s Republic of China; 5 Key Laboratory of Ministry of Education for Gastrointestinal Cancer, Fujian Medical University, Fuzhou, People’s Republic of China

**Keywords:** Gastrointestinal cancer, Surgical oncology

## Abstract

Previous studies indicate differences in short-term postoperative outcomes depending on the surgical starting time of the day, but long-term data are lacking. The aim of this study was to clarify if surgical starting time of the day influences long-term survival in gastric cancer patients. This cohort study consecutively included 2728 patients who underwent curatively intended gastrectomy for gastric cancer in 2011–2015 at a high-volume hospital in China, with follow-up until June 2019. Cox regression provided hazard ratios (HRs) with 95% confidence intervals (CIs) for 3-year all-cause mortality, adjusted for age, sex, health insurance, pathological tumor stage, surgical approach, neoadjuvant therapy, and weekday of surgery. Compared with patients with early starting time of gastrectomy (08:00–09:29), the point estimates for 3-year all-cause mortality were modestly increased in patients with a starting time in the middle of day (09:30–13:29; HR 1.15, 95% CI 0.97 to 1.37) and later (13:30–21:25; HR 1.10, 0.91 to 1.32). The corresponding HRs were increased particularly in patients who underwent laparoscopic gastrectomy (HR 1.54, 1.10 to 2.14 and HR 1.59, 1.12 to 2.25, respectively) and in those with stage II tumors (HR 1.74, 1.11 to 2.73 and HR 1.60, 1.00 to 2.58, respectively). Our study indicated that in patients who underwent laparoscopic gastrectomy and in those who with stage II tumors, starting surgery in the early morning might be associated with better long-term survival.

## Introduction

Gastric cancer is the fifth most common cancer and the fourth leading cause of cancer-related death worldwide, with an annual incidence of over one million new cases and nearly 800,000 deaths^1^. The rates are highest in East Asia (42% of all cases worldwide occurring in China), East Europe, and South America^2^. The 5-year overall survival in gastric cancer is below 40%^3^. Surgical resection with total or subtotal gastrectomy is the main curatively intended treatment. Optimization of this surgery would improve the survival.

‘Time of day’ variations in various interventions may influence short-term outcomes in several diseases^4–6^, including cancer^7,8^. Some surgeons in high-volume centers perform two or three major surgeries each day^9,10^, although the procedures are demanding and time-consuming, requiring high surgical skills and concentration^11^. Whether ‘time of day’ variation in gastrectomy influences long-term survival in gastric cancer is unknown and evidence of how this influences short-term outcomes is scarce^12^. Interestingly, some studies have found increased postoperative mortality in patients who undergo various types of surgery later as opposed to earlier in the week^13–15^. Some research specifically suggests that later weekday of gastrectomy independent of other prognostic factors increases the long-term disease-specific mortality in gastric cancer^16^. A possible explanation is a negative influence of the cumulative workload during the working week. We hypothesized that similar mechanisms exist for the timing of gastrectomy during the day, resulting in worse long-term survival and short-term outcomes in gastric cancer patients if the procedure is conducted later in the day. This hypothesis was examined in a cohort study from a high-volume center of gastrectomy in China.

## Methods

### Design

**Figure 1:**
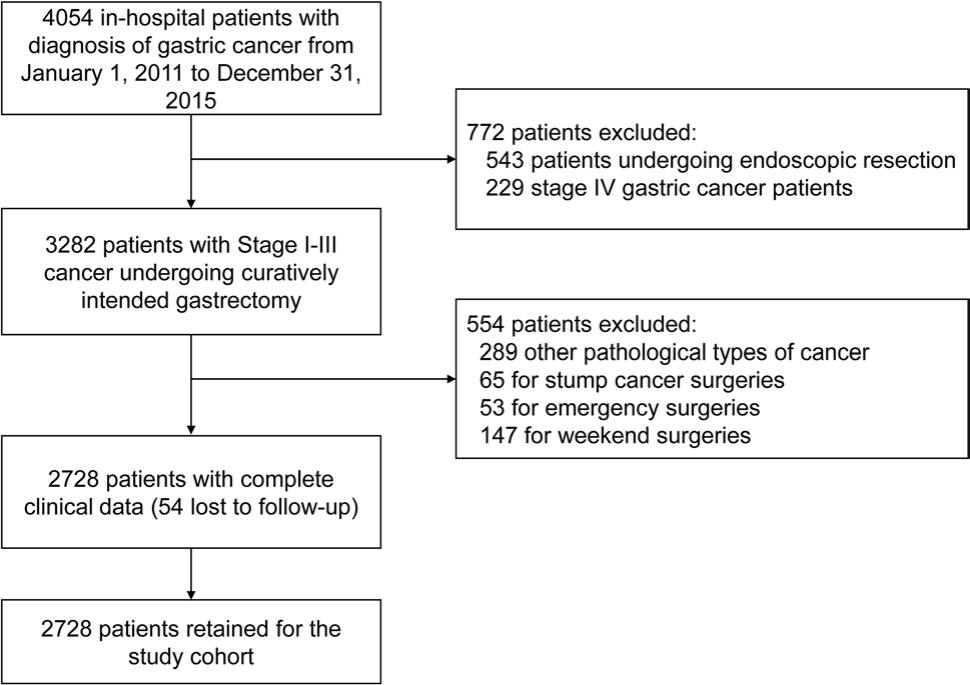
Flow chart of patient enrollment in this study.

This cohort study consecutively included all patients who underwent total or subtotal gastrectomy for gastric cancer in a cancer center in Beijing, China between January 1, 2011 and December 31, 2015, with follow-up until June 30, 2019. The start date was chosen because minimally invasive surgery was broadly used in this hospital from 2011 onwards. An earlier version of this cohort has been described elsewhere^17^. The following patients were eligible for the cohort: (1) age ≥ 18 years, (2) histologically confirmed primary gastric adenocarcinoma, (3) pathological tumor stage I-III according to the American Joint Committee on Cancer TNM staging system, 7th edition^18^, (4) curatively intended gastrectomy, and (5) planned gastrectomy performed during the working week (Monday to Friday). Patients were excluded if they had (1) emergency gastrectomy, (2) palliative gastrectomy, (3) gastric stump cancer, or (4) other histological gastric tumor types than adenocarcinoma, (5) gastrectomies performed in weekends (Fig. 1).

This study was performed in accordance with the Declaration of Helsinki. The institutional review board of Chinese PLA General Hospital approved this retrospective study (reference number, S2019-040-01), in which patient informed consent from individual participant was waived due to retrospective nature and anonymous process of this study.

### Data collection

Data on the baseline characteristics of patients, including sex, age, hospital stays, comorbidity, and health insurance, were extracted directly from the electronic medical records (EMR) system held by the Department of Medical Big Data in the cancer center. The surgical and post-operative details were independently reviewed by two researchers (WL and HX), who were blinded to the study hypothesis. Comorbidity was assessed using the well-validated Charlson comorbidity index^19^, and the insurance coverage was used as an indicator for socioeconomic status. The study followed the Strengthening the Reporting of Observational Studies in Epidemiology (STROBE) guidelines^20^.

### Exposure

The study exposure was the starting time of first incision for gastrectomy, which was extracted as a structural characteristic from the anesthesiology charts in the EMR system. Patients received curatively-intended gastrectomies by a total of 16 senior surgeon teams who were trained and qualified for both open and minimally invasive gastrectomy. The distribution of surgical starting time is displayed in supplemental Fig. S1. The starting time range was 08:00 to 21:25. The patients were categorized into three approximately equal-sized groups (tertiles) according to surgical starting time: an early morning group with starting time between 08:00 and 09:29 (N = 929), an intermediate group with starting time between 09:30 and 13:29 (N = 955), and a late group with starting time between 13:30 and 21:25 (N = 844). To further minimize selection bias, analysis by four approximately equal time interval groups (08:00–11:00, 11:00–14:00, 14:00–17:00, 17:00-after) was also performed (Table S1).

### Outcomes

The primary outcome was 3-year all-cause mortality. The 3-year cut-off was chosen instead of 5 years for three reasons: (1) The 3-year mortality mirrors longer term survival, (2) the clinical follow-up practice in the center, and (3) it was possible to follow all patients in the cohort for 3 years within the study period. Data on 3-year all-cause mortality were collected from the patients’ medical records or via telephone follow-up (every 3 months for the first 2 years and every 6 months the third year). Secondary outcomes were total number of retrieved lymph nodes and length of postoperative hospital stay. These data were extracted directly from the medical records and pathology reports.

### Statistical analysis

The patients were followed up from the date of gastrectomy until the end of study or death, whichever occurred first. The association between surgical starting time of the day and 3-year all-cause mortality was assessed using multivariable Cox proportional hazards regression, providing hazard ratios (HRs) with 95% confidence intervals (CIs). Seven predefined covariates were included in a multivariable model because of their known prognostic influence in combination with possible influence on the surgical starting time of the day: (1) age at surgery (continuous), (2) sex (male or female), (3) health insurance coverage (yes or no), (4) neoadjuvant therapy (yes or no), (5) pathological tumor stage (0-I, II, or III), and (6) surgical approach (open, laparoscopic or robotic), and (7) weekday of surgery (Monday-Wednesday or Thursday-Friday). The proportional hazards assumption was tested by Schoenfeld residuals and was met in all analyses. To explore associations within specific subgroups, analyses were stratified by the aforementioned 7 covariates using the same categorization, as well as by Charlson comorbidity (0, 1, or ≥ 2) and tumor location (cardia or non-cardia). Survival curves for different surgical starting time groups were generated using Kaplan–Meier estimates and compared by the log-rank test.

The secondary outcomes, i.e. postoperative stay and number of retrieved lymph nodes, were both treated as binary variables with the cut-off set at the median values, i.e. 11 days of postoperative stay and 23 lymph nodes. The association between surgical starting time of the day and these outcomes was assessed using multivariable logistic regression, providing odds ratios (ORs) with 95% CIs, adjusted for the seven covariates (with the same categorization) presented above.

All statistical analyses followed a detailed pre-defined study protocol and were performed by first author (YG) and checked by an experienced statistician (FM) using the SPSS software, version 25 (SPSS Inc, Chicago, IL). All tests were 2-sided and statistical significance was set at *P* < 0.05.

## Results

### Patients

A total of 2728 patients who underwent planned and curatively intended gastrectomy for gastric cancer were included in the cohort. Most patients were men (2065, 75.7%) and the median age was 60 years (interquartile range 52–67). There were no major differences in the distribution of age, sex, tumor location, pathological tumor stage, comorbidity, or neoadjuvant therapy among the three surgical starting time groups, but there was a trend of more self-paid patients in the two later surgical starting time groups compared to the early group (Table 1). The distribution of surgical approach, reconstruction method, and extent of the lymph node dissection was similar in the three surgical starting time groups (Table 2). The patients were followed up for a median of 50 months (interquartile range 29–73). Among all participants, 54 (2%) were lost to follow-up and were censored.

**Table 1:** Characteristics of 2728 patients who underwent gastrectomy for gastric cancer, number (%).

Variable	Surgical starting time during the day			
	08:00–09:29	09:30–13:29	13:30–21:25	*p* value
Total	929 (100.0)	955 (100.0)	844 (100.0)	
Age, years (median, IQR)	60.0 (52.0–68.0)	61.0 (52.0–68.0)	60.0 (51.0–66.0)	0.063
Sex				0.681
Men	695 (74.8)	731 (75.7)	639 (75.7)	
Women	234 (25.2)	224 (24.3)	205 (24.3)	
Gastric cancer location				0.342
Cardia	327 (35.2)	386 (40.4)	319 (37.8)	
Non-cardia				
Body	139 (15.0)	136 (14.2)	116 (13.7)	
Antrum	441 (47.5)	413 (43.2)	385 (45.6)	
Whole	22 (2.4)	20 (2.1)	24 (2.8)	
Pathological tumor stage^a^				0.738
I	299 (32.2)	286 (29.9)	255 (30.2)	
II	252 (27.1)	253 (26.5)	226 (26.8)	
III	378 (40.7)	416 (43.6)	363 (43.0)	
Differentiation grade				0.695
Well	48 (5.2)	47 (4.9)	56 (6.6)	
Moderate	174 (18.7)	173 (18.1)	162 (19.2)	
Moderate-to-poor	249 (26.8)	263 (27.5)	214 (25.4)	
Poor	458 (49.3)	472 (49.5)	412 (48.8)	
Charlson comorbidity score				0.360
0	158 (17.0)	159 (16.7)	138 (16.4)	
1	238 (25.6)	239 (25.0)	245 (29.0)	
≥ 2	533 (57.4)	557 (58.3)	461 (54.6)	
Neoadjuvant therapy				0.028
No	854 (91.9)	904 (94.7)	797 (94.4)	
Yes	75 (8.1)	51 (5.3)	47 (5.6)	
Health insurance coverage				< 0.001
No	695 (74.8)	738 (77.3)	719 (85.2)	
Yes	234 (25.2)	217 (22.7)	125 (14.8)	

*IQR* interquartile range.

^a^Pathological tumor stage according to the American Joint Committee on Cancer TNM staging system, 7th edition.

**Table 2:** Surgical and postoperative variables among 2728 patients who underwent gastrectomy for gastric cancer, number (%).

Variable	Surgical starting time of the day			
	08:00–09:29	09:30–13:29	13:30–21:25	*p* value
Gastrectomy				0.249
Proximal	259 (27.9)	305 (31.9)	233 (27.6)	
Distal	449 (48.3)	441 (46.2)	410 (48.6)	
Total	221 (23.8)	209 (21.9)	201 (23.8)	
Surgical approach				< 0.001
Open	573 (61.7)	559 (58.5)	543 (64.3)	
Laparoscopic	276 (29.7)	328 (34.4)	273 (32.4)	
Robotic	80 (8.6)	68 (7.1)	28 (3.3)	
Reconstruction				0.070
B-I	220 (23.7)	216 (22.6)	201 (23.8)	
B-II	183 (19.7)	207 (21.7)	190 (22.5)	
Roux-en-Y	279 (30.0)	234 (24.5)	222 (26.3)	
Others	247 (26.6)	298 (31.2)	231 (27.4)	
Operation duration, minutes (mean ± SD)	213.3 ± 58.6	210.5 ± 62.4	208.5 ± 59.6	0.239
Lymph node dissection				0.161
D1/D1 +	595 (64.0)	649 (68.0)	546 (64.7)	
D2	334 (36.0)	306 (32.0)	298 (35.3)	
Lymph node retrieval (mean ± SD)	25.4 ± 12.0	24.8 ± 11.9	25.7 ± 12.4	0.241
Post-operative hospital stay, days (mean ± SD)	13.3 ± 7.9	13.3 ± 8.5	13.1 ± 10.2	0.920
Post-operative complication (Clavien-Dindo)				0.803^a^
I	37 (4.0)	30 (3.1)	37 (4.4)	
II	47 (5.1)	42 (4.4)	33 (3.9)	
III	14 (1.5)	10 (1.0)	10 (1.2)	
IV	5 (0.5)	4 (0.4)	6 (0.7)	
V	1 (0.1)	1 (0.1)	0 (0)	

*SD* standard deviation.

^a^Fisher’s exact test.

### 3-year all-cause mortality

**Figure 2:**
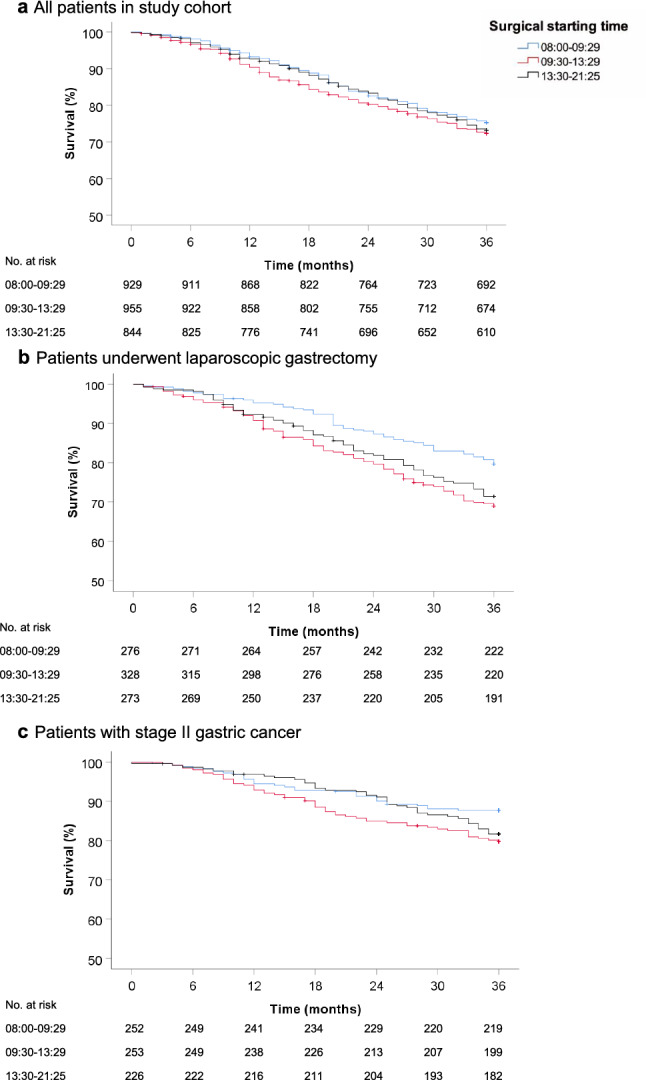
Kaplan–Meier overall survival curves in gastric cancer patients who underwent gastrectomy by surgical starting time of the day: (**a**) All patients; (**b**) Patients who had undergone laparoscopic gastrectomy; (**c**) Patients with stage II gastric cancer.

The 3-year all-cause mortality rate was 26.0% in the entire cohort and no major differences were observed among the three surgical starting time groups in crude analysis (Fig. 2a, log-rank test, *P* = 0.269). Compared to the early starting group, the adjusted point estimates for the intermediate starting time group (HR 1.15, 95% CI 0.97 to 1.37) and the late starting group (HR 1.10, 0.91 to 1.32) were increased, but not statistically significant (Table 3). In patients who underwent laparoscopic gastrectomy, the adjusted HRs were increased in the intermediate starting time group (HR 1.54, 1.10 to 2.14) and in the late group (HR 1.59, 1.12 to 2.25), compared to the early starting group (Table 3 and Fig. 2b). A similar trend was suggested in patients who had undergone robotic surgery in late starting group (HR 1.36, 0.58 to 3.17), although the estimates were not statistically significant. No such association was indicated for open surgery. In patients diagnosed with pathological tumor stage II, the 3-year all-cause mortality was increased in the intermediate starting time group (HR 1.74, 1.11 to 2.73) and in the late group (HR 1.60, 1.00 to 2.58), compared to the early group (Table 3 and Fig. 2c). No association between gastrectomy starting time and 3-year all-cause mortality was found in patients diagnosed with early (I) or advanced pathological tumor stage (III). No clear differences in associations were found for subgroups of sex, age, comorbidity, tumor location, health insurance coverage, neoadjuvant therapy, or weekday of surgery, although almost all point estimates were above 1.0 in the two later starting time groups compared to the first (Table 3).

**Table 3:** Surgical starting time of the day for gastric cancer surgery in relation to 3-year all-cause mortality, presented as hazard ratios (HRs) with 95% confidence intervals (CIs).

Variable	Patients Number (%)	Deaths Number (%)	HR (95% CI) by surgical starting time^a^		
			08:00–09:29	09:30–13:29	13:30–21:25
Total	2728 (100.0)	710 (26.0)	1 (reference)	1.15 (0.97–1.37)	1.10 (0.91–1.32)
Sex					
Male	2065 (75.7)	542 (26.2)	1 (reference)	1.11 (0.90–1.36)	1.21 (0.98–1.50)
Female	663 (24.3)	168 (25.3)	1 (reference)	1.33 (0.93–1.91)	0.85 (0.57–1.26)
Age, years					
≤ 60	1394 (51.1)	281(20.2)	1 (reference)	1.24 (0.93–1.65)	1.10 (0.81–1.49)
> 60	1334 (48.9)	429 (32.2)	1 (reference)	1.06 (0.84–1.33)	1.07 (0.84–1.36)
Charlson comorbidity score					
0	455 (16.7)	93 (20.4)	1 (reference)	1.31 (0.81–2.13)	0.85 (0.50–1.46)
1	722 (26.5)	142 (19.7)	1 (reference)	1.52 (0.98–2.33)	1.35 (0.86–2.10)
≥ 2	1551 (56.9)	475 (30.6)	1 (reference)	1.05 (0.84–1.30)	1.13 (0.90–1.42)
Surgical approach					
Open	1675 (61.4)	437 (26.1)	1 (reference)	1.01 (0.80–1.27)	0.92 (0.73–1.17)
Laparoscopic	877 (32.1)	233 (26.6)	1 (reference)	1.54 (1.10–2.14)	1.59 (1.12–2.25)
Robotic	176 (6.5)	40 (21.7)	1 (reference)	0.91 (0.42–1.98)	1.36 (0.58–3.17)
Pathological tumor stage					
I	840 (30.8)	48 (5.7)	1 (reference)	0.89 (0.45–1.76)	0.95 (0.47–1.93)
II	731 (26.8)	123 (16.8)	1 (reference)	1.74 (1.11–2.73)	1.60 (1.00–2.58)
III	1157 (42.4)	539 (46.6)	1 (reference)	1.08 (0.88–1.33)	1.05 (0.85–1.30)
Tumor location^b^					
Cardia	1033 (37.9)	284 (27.5)	1 (reference)	1.21 (0.91–1.61)	1.05(0.77–1.43)
Non-cardia	1629 (59.7)	386 (23.7)	1 (reference)	1.11 (0.87–1.42)	1.14 (0.89–1.47)
Neoadjuvant therapy					
Yes	173 (6.3)	42 (26.0)	1 (reference)	1.07 (0.49–2.34)	1.35 (0.63–2.92)
No	2555 (93.7)	668 (26.1)	1 (reference)	1.14 (0.95–1.37)	1.10 (0.90–1.33)
Weekday of surgery					
Monday–Wednesday	1698 (62.2)	444 (26.1)	1 (reference)	1.22 (0.97–1.54)	1.09 (0.86–1.38)
Thursday–Friday	1030 (37.8)	266 (25.8)	1 (reference)	1.05 (0.79–1.39)	1.15 (0.84–1.57)
Health insurance coverage					
Yes	576 (21.1)	161(28.0)	1 (reference)	1.40 (0.96–2.04)	1.40 (0.94–2.10)
No	2152 (78.9)	549 (25.5)	1 (reference)	1.05 (0.85–1.29)	1.00 (0.81–1.24)

^a^Adjusted for age, sex, health insurance coverage, neoadjuvant therapy, pathological tumor stage, surgical approach, weekday of surgery.

^b^66 cases with tumors affecting multiple anatomical locations were not included in this analysis.

In analysis by quartile of time groups (Table S1), subgroup analysis also demonstrated that the 3-year all-cause mortality was increased in intermediate time group (14:00–17:00, HR 1.55, 95%CI 1.10–2.18) compared to early starting time group (08:00–11:00) in laparoscopic approach patients. Similarly, in patients with stage II gastric cancer, elevated mortality was observed in intermediate (11:00–14:00, HR 1.93, 1.25–2.97) and late starting time (17:00-after, HR 2.15, 1.00–4.64) groups.

### Lymph node retrieval and length of postoperative stay

Gastrectomy performed later during the day was not associated with obvious increased odds of lymph node retrieval (Table 4, OR 0.88, 95% CI 0.73 to 1.06 for 09:30–13:29 and OR 1.06, 0.88 to 1.28 for 13:30–21:25) or length of postoperative stay (OR 0.98, 0.81 to 1.17 for 09:30–13:29 and OR 0.83, 0.69 to 1.01 for 13:30–21:25) compared to early starting time (08:00–09:29).

**Table 4:** Surgical starting time of the day for gastric cancer surgery in relation to lymph node retrieval and length of postoperative stay.

Starting time	Patients number (%)	Odds ratio (95% confidence interval)^a^	
		Lymph node yield	Postoperative stay
08:00–09:29	929 (34.1)	1 (reference)	1 (reference)
09:30–13:29	955 (35.0)	0.88 (0.73–1.06)	0.98 (0.81–1.17)
13:30–21:25	844 (30.9)	1.06 (0.88–1.28)	0.83 (0.68–1.01)

^a^Adjusted for age, sex, health insurance coverage, neoadjuvant therapy, pathological tumor stage, surgical approach, weekday of surgery.

## Discussion

This study indicated an increased risk of 3-year all-cause mortality in gastric cancer patients if the surgical starting time was later in the day than in the early morning, particularly in those who underwent laparoscopic gastrectomy and with pathological stage II tumor. The surgical starting time during the day did not influence the lymph node yield or length of postoperative hospital stay.

Some methodological issues need to be discussed in order to interpret the findings. First, it was not feasible to randomly assign the surgical starting time of the day, which left us with an observational design. Second, this study was based on one of the largest cancer centers in China and focused on gastric cancer, thus providing a large sample size and counteracting disease heterogeneity. The single-center high-volume approach also allowed complete and detailed clinical data and at least partly counteracted bias resulting from different surgeon volumes. On the other hand, the results from this single center might be less generalizable. Third, the assessment of the gastrectomy starting time (exposure), 3-year all-cause mortality (main outcome) and covariates was objective and accurate. The surgery day rotation system at the center enabled each consultant surgeon to have similar opportunities to arrange their operation schedule, meaning that factors like age or experience of the surgeon would not influence the surgical starting time. However, due to lack of data on cause-specific death, we could not assess disease-specific mortality. Fourth, potential confounding by the main prognostic factors was carefully adjusted for in the analyses, but residual confounding cannot be ruled out.

To the best of our knowledge, this is the first study examining the role of ‘time of the day’ variations in surgery in relation to long-term survival in gastric cancer. The finding that gastric cancer patients who underwent laparoscopic surgery and those diagnosed with pathological tumor stage II tended to have worse survival if the gastrectomy was started later in the day is interesting. Chance cannot be excluded as an explanation, but the findings may also be true. Speculatively, the workload accumulation during the day might influence the performance of the surgeons and the surgical team^21,22^. This could be more of an issue for laparoscopic surgery than for open surgery, because laparoscopic procedures tend to be more time-consuming and technically demanding^23,24^. However, statistically differences were not observed in robotic assisted gastrectomy. We speculated that limited sample size of robotic surgery might mainly account for this finding, which calls for further studies with larger cohort of robotic surgeries to address. A possible explanation for the tumor stage II-specific finding is a that the fine-tuning of the surgical accuracy may be less critical in patients with earlier tumor stage who usually have a very high survival rate anyway, while those with more advanced tumors more often have invisible tumor spread beyond surgical cure^25,26^. Patients with stage II tumors, on the other hand, may benefit most from the best possible surgical treatment. The absence of better survival in the middle starting group than that the last starting group may speculatively be due to the fact that surgeons did not get a break or any food before starting surgery around lunch time.

Because lymphadenectomy^27,28^ and postoperative complications and re-operations might influence the long-term survival^29,30^, we explored the surgical starting time during the day in relation to lymph node retrieval and length of hospital stay. The lack of associations with these outcomes indicate that these factors were not mediators of the worse survival in gastric cancer patients who underwent surgery later in the day.

The findings from this first study examining the topic need confirmation in future research before any clinical implications can be considered. Large population-based studies examining a more detailed grouping of the surgical starting time may be particularly useful in this respect. If proven true, these results indicate a need to tailor the starting time of gastrectomy. Although this study focused on surgery for gastric cancer, it is possible that similar mechanism and results might be generalizable to other challenging surgical cancer procedures, for example, surgery for colorectal, hepatobiliary and pancreatic cancers.

## Conclusions

In conclusion, this current study indicated that in patients with gastric cancer, especially those who undergo laparoscopic gastrectomy and those diagnosed with stage II tumors, initiating surgery in the early morning was possibly associated with better prognosis, which still needs further prospective clinical trials to verify.

## Supplementary Information

Supplementary Figure S1.

Supplementary Table S1.

## Data Availability

The anonymous data during the current study are available from the corresponding author upon reasonable request.

## References

[1] Sung H (2021). Global cancer statistics 2020: GLOBOCAN estimates of incidence and mortality worldwide for 36 cancers in 185 countries. CA Cancer J. Clin..

[2] Chen W (2016). Cancer statistics in China, 2015. CA Cancer J. Clin..

[3] Allemani C (2015). Global surveillance of cancer survival 1995–2009: Analysis of individual data for 25,676,887 patients from 279 population-based registries in 67 countries (CONCORD-2). Lancet.

[4] Kelz RR (2008). Time of day is associated with postoperative morbidity: An analysis of the national surgical quality improvement program data. Ann. Surg..

[5] Magid DJ (2005). Relationship between time of day, day of week, timeliness of reperfusion, and in-hospital mortality for patients with acute ST-segment elevation myocardial infarction. JAMA.

[6] Uusaro A, Kari A, Ruokonen E (2003). The effects of ICU admission and discharge times on mortality in Finland. Intensive Care Med..

[7] Ishiyama Y (2019). Surgical starting time in the morning versus the afternoon: Propensity score matched analysis of operative outcomes following laparoscopic colectomy for colorectal cancer. Surg. Endosc..

[8] Slaughter KN (2014). Minimally invasive surgery for endometrial cancer: Does operative start time impact surgical and oncologic outcomes. Gynecol. Oncol..

[9] Choi YY (2019). Ten thousand consecutive gastrectomies for gastric cancer: Perspectives of a master surgeon. Yonsei Med. J..

[10] Kim EY, Song KY, Lee J (2017). Does hospital volume really affect the surgical and oncological outcomes of gastric cancer in Korea. J. Gastric Cancer.

[11] Brenkman HJ, Haverkamp L, Ruurda JP, van Hillegersberg R (2016). Worldwide practice in gastric cancer surgery. World J. Gastroenterol..

[12] Sessler DI, Kurz A, Saager L, Dalton JE (2011). Operation timing and 30-day mortality after elective general surgery. Anesth. Analg..

[13] Aylin P, Alexandrescu R, Jen MH, Mayer EK, Bottle A (2013). Day of week of procedure and 30 day mortality for elective surgery: Retrospective analysis of hospital episode statistics. BMJ.

[14] Lagergren J, Mattsson F, Lagergren P (2016). Weekday of esophageal cancer surgery and its relation to prognosis. Ann. Surg..

[15] Zare MM, Itani KM, Schifftner TL, Henderson WG, Khuri SF (2007). Mortality after nonemergent major surgery performed on Friday versus Monday through Wednesday. Ann. Surg..

[16] Lagergren J, Mattsson F, Lagergren P (2017). Weekday of cancer surgery in relation to prognosis. Br. J. Surg..

[17] Gao Y (2019). Comparison of robotic- and laparoscopic-assisted gastrectomy in advanced gastric cancer: Updated short- and long-term results. Surg. Endosc..

[18] Washington K (2010). 7th edition of the AJCC cancer staging manual: stomach. Ann. Surg. Oncol..

[19] Charlson ME, Pompei P, Ales KL, MacKenzie CR (1987). A new method of classifying prognostic comorbidity in longitudinal studies: Development and validation. J. Chronic Dis..

[20] von Elm E (2007). The strengthening the reporting of observational studies in epidemiology (STROBE) statement: Guidelines for reporting observational studies. PLoS Med..

[21] Eastridge BJ (2003). Effect of sleep deprivation on the performance of simulated laparoscopic surgical skill. Am. J. Surg..

[22] Gawande AA, Zinner MJ, Studdert DM, Brennan TA (2003). Analysis of errors reported by surgeons at three teaching hospitals. Surgery.

[23] Berguer R, Smith WD, Chung YH (2001). Performing laparoscopic surgery is significantly more stressful for the surgeon than open surgery. Surg. Endosc..

[24] Lee HJ (2019). Short-term outcomes of a multicenter randomized controlled trial comparing laparoscopic distal gastrectomy with D2 lymphadenectomy to open distal gastrectomy for locally advanced gastric cancer (KLASS-02-RCT). Ann. Surg..

[25] Nashimoto A (2013). Gastric cancer treated in 2002 in Japan: 2009 annual report of the JGCA nationwide registry. Gastric Cancer.

[26] Sano T (2017). Proposal of a new stage grouping of gastric cancer for TNM classification: International Gastric Cancer Association staging project. Gastric Cancer.

[27] Ajani JA (2016). Gastric cancer, version 3.2016, NCCN clinical practice guidelines in oncology. J. Natl. Compr. Cancer Netw..

[28] Japanese Gastric Cancer Association (2011). Japanese classification of gastric carcinoma: 3rd English edition. Gastric Cancer.

[29] Gertsen EC, Goense L, Brenkman H, van Hillegersberg R, Ruurda JP, Dutch Upper Gastrointestinal Cancer Audit (DUCA) Group (2019). Identification of the clinically most relevant postoperative complications after gastrectomy: a population-based cohort study. Gastric Cancer.

[30] Raziee HR (2012). Systematic review of the predictors of positive margins in gastric cancer surgery and the effect on survival. Gastric Cancer.

