# Supplementary material for: Surgical starting time of the day and survival in gastric cancer
Source: Sci Rep. 2023 Apr 28;13:6955. doi: 10.1038/s41598-023-33692-0 (PMC10147916; doi:10.1038/s41598-023-33692-0)
Supplement: Supplementary file 1 — Supplementary Figure S1. [file 41598_2023_33692_MOESM1_ESM.pdf]

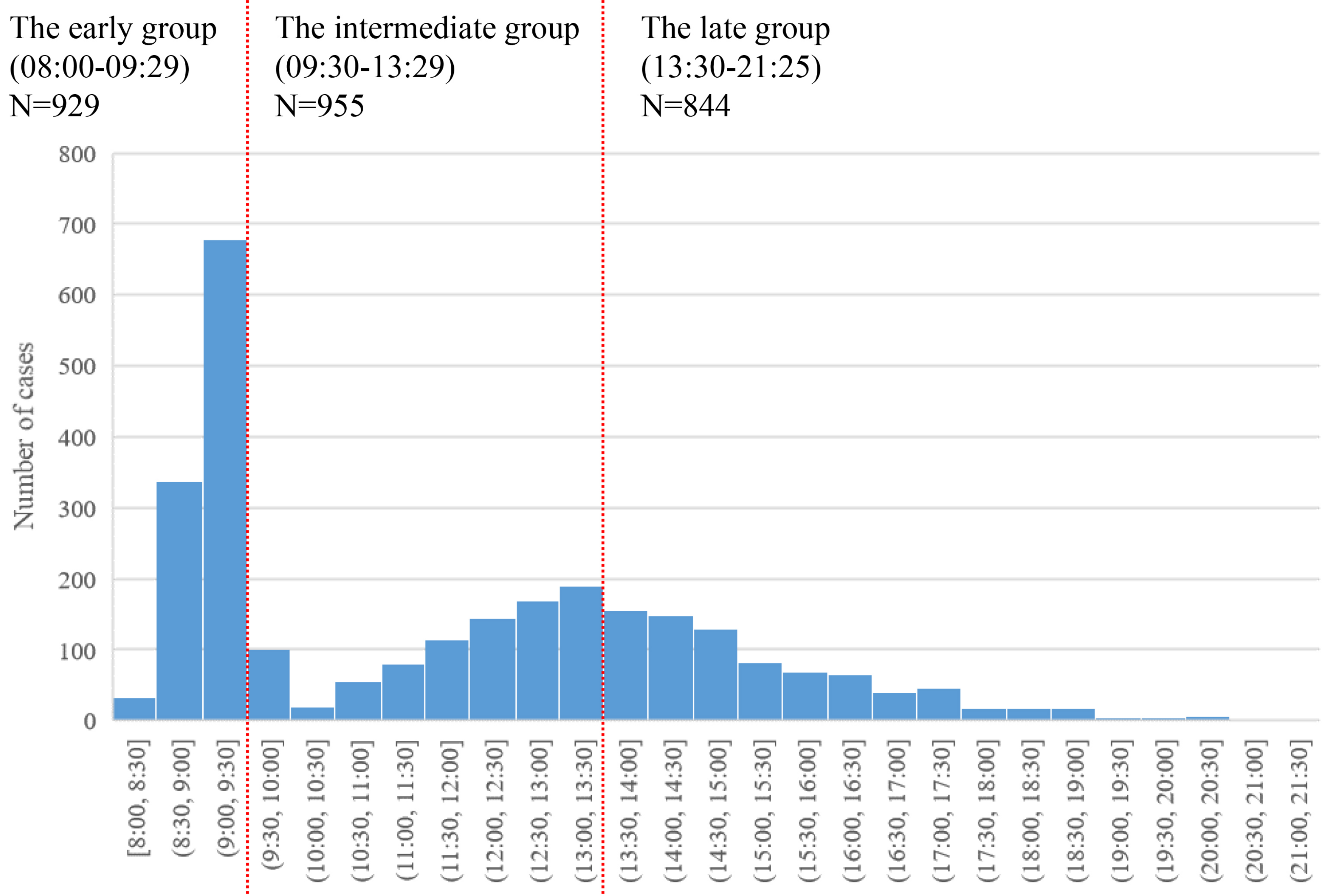

Figure S1. Distribution of surgical starting time of the day in 2728 patients who underwent gastrectomy for gastric cancer.
