# Supplementary material for: Surgical starting time of the day and survival in gastric cancer
Source: Sci Rep. 2023 Apr 28;13:6955. doi: 10.1038/s41598-023-33692-0 (PMC10147916; doi:10.1038/s41598-023-33692-0)
Supplement: Supplementary file 2 — Supplementary Table S1. [file 41598_2023_33692_MOESM2_ESM.pdf]

Table S1. Surgical starting time (quartiles) of the day for gastric cancer surgery in relation to 3-year all-cause mortality, presented as hazard ratios (HRs) with 95% confidence intervals (CIs).

Variable	Patients Number (%)	HR (95% CI) by surgical starting time[a]			
		**08:00-11:00 (1206, 44.2%)**	**11:00-14:00 (837, 30.7%)**	**14:00-17:00 (564, 20.7%)**	**17:00-after (121, 4.4%)**
Total	2728 (100.0)	1 (reference)	1.07 (0.90-1.28)	1.15 (0.93-1.40)	1.02 (0.71-1.46)
Sex					
Male	2065 (75.7)	1 (reference)	1.07 (0.87-1.31)	1.23 (0.98-1.55)	1.08 (0.73-1.59)
Female	663 (24.3)	1 (reference)	1.14 (0.79-1.64)	0.92 (0.61-1.38)	0.81 (0.29-2.23)
Age, years					
≤60	1394 (51.1)	1 (reference)	1.00 (0.79-1.33)	1.01 (0.74-1.38)	0.83 (0.45-1.55)
>60	1334 (48.9)	1 (reference)	1.08 (0.86-1.35)	1.19 (0.92-1.54)	1.08 (0.69-1.68)
Charlson comorbidity score					
0	455 (16.7)	1 (reference)	1.13 (0.70-1.84)	0.91 (0.53-1.56)	0.66 (0.16-2.75)
1	722 (26.5)	1 (reference)	1.16 (0.78-1.72)	1.01 (0.64-1.60)	1.10 (0.47-2.58)
≥2	1551 (56.9)	1 (reference)	1.05 (0.85-1.31)	1.24 (0.97-1.58)	1.16 (0.76-1.77)
Surgical approach					
Open	1675 (61.4)	1 (reference)	1.06 (0.85-1.32)	0.97 (0.75-1.25)	0.98 (0.60-1.59)
Laparoscopic	877 (32.1)	1 (reference)	1.07 (0.78-1.46)	**1.55 (1.10-2.18)**	1.09 (0.63-1.88)
Robotic	176 (6.5)	1 (reference)	1.51 (0.64-3.58)	1.25 (0.46-3.36)	/ [c]
Pathological tumor stage					
I	840 (30.8)	1 (reference)	0.64 (0.31-1.31)	0.86 (0.40-1.84)	0.71 (0.10-5.39)
II	731 (26.8)	1 (reference)	**1.93 (1.25-2.97)**	1.48 (0.88-2.48)	**2.15 (1.00-4.64)**
III	1157 (42.4)	1 (reference)	0.99 (0.81-1.21)	1.12 (0.89-1.41)	0.90 (0.59-1.37)
Tumor location[b]					
Cardia	1033 (37.9)	1 (reference)	0.70 (0.43-1.15)	0.64 (0.38-1.08)	0.52 (0.16-1.68)
Non-cardia	1629 (59.7)	1 (reference)	1.40 (0.95-2.04)	**1.74 (1.14-2.64)**	1.40 (0.66-2.96)
Neoadjuvant therapy					
Yes	173 (6.3)	1 (reference)	0.81 (0.36-1.85)	0.91 (0.36-2.28)	4.86 (1.56-15.16)
No	2555 (93.7)	1 (reference)	1.09 (0.91-1.31)	1.16 (0.94-1.42)	0.90 (0.61-1.33)
Weekday of surgery					
Monday-Wednesday	1698 (62.2)	1 (reference)	1.15 (0.91-1.44)	1.11 (0.87-1.43)	1.22 (0.80-1.87)
Thursday-Friday	1030 (37.8)	1 (reference)	0.97 (0.73-1.29)	1.24 (0.88-1.74)	0.67 (0.33-1.38)
Health insurance coverage					
Yes	576 (21.1)	1 (reference)	1.25 (0.86-1.82)	1.29 (0.81-2.05)	0.92 (0.42-2.01)
No	2152 (78.9)	1 (reference)	1.02 (0.84-1.24)	1.07 (0.86-1.34)	1.03 (0.68-1.55)

[a] Adjusted for age, sex, health insurance coverage, neoadjuvant therapy, pathological tumor stage, surgical approach, weekday of surgery.

[b]66 cases with tumors affecting multiple anatomical locations were not included in this analysis.

[c]No robotic surgery was performed in the late starting time group
